# Socioeconomic disparities in head and neck cancer survival in Germany: a causal mediation analysis using population-based cancer registry data

**DOI:** 10.1007/s00432-021-03537-2

**Published:** 2021-02-11

**Authors:** Ahmed Bedir, Semaw Ferede Abera, Ljupcho Efremov, Lamiaa Hassan, Dirk Vordermark, Daniel Medenwald

**Affiliations:** 1 Department of Radiation Oncology, Health Services Research Group, University Hospital Halle (Saale), Ernst-Grube-Str. 40, 06120 Halle (Saale), Germany; 2 Institute of Medical Epidemiology, Biometry, and Informatics, Martin Luther University Halle-Wittenberg, Magdeburger Strasse 8, 06112 Halle (Saale), Germany; 3 Department of Radiation Oncology, University Hospital Halle (Saale), Ernst-Grube-Str. 40, 06120 Halle (Saale), Germany

**Keywords:** Head and neck cancer, Survival, Socioeconomic deprivation, Causality, Mediation analysis

## Abstract

**Purpose:**

Despite recent improvements in cancer treatment in Germany, a marked difference in cancer survival based on socioeconomic factors persists. We aim to quantify the effect of socioeconomic inequality on head and neck cancer (HNC) survival.

**Methods:**

Information on 20,821 HNC patients diagnosed in 2009–2013 was routinely collected by German population-based cancer registries. Socioeconomic inequality was defined by the German Index of Socioeconomic Deprivation. The Cox proportional regression and relative survival analysis measured the survival disparity according to level of socioeconomic deprivation with respective confidence intervals (CI). A causal mediation analysis was conducted to quantify the effect of socioeconomic deprivation mediated through medical care, stage at diagnosis, and treatment on HNC survival.

**Results:**

The most socioeconomically deprived patients were found to have the highest hazard of dying when compared to the most affluent (Hazard Ratio: 1.25, 95% CI 1.17–1.34). The most deprived patients also had the worst 5-year age-adjusted relative survival (50.8%, 95% CI 48.5–53.0). Our mediation analysis showed that most of the effect of deprivation on survival was mediated through differential stage at diagnosis during the first 6 months after HNC diagnosis. As follow-up time increased, medical care, stage at diagnosis, and treatment played no role in mediating the effect of deprivation on survival.

**Conclusion:**

This study confirms the survival disparity between affluent and deprived HNC patients in Germany. Considering data limitations, our results suggest that, within six months after HNC diagnosis, the elimination of differences in stage at diagnosis could reduce survival inequalities.

**Supplementary Information:**

The online version contains supplementary material available at 10.1007/s00432-021-03537-2.

## Introduction

Head and neck cancer (HNC) accounts for approximately 3% of all new malignancies in Germany, and is ranked the seventh most common cancer worldwide (Global Burden of Disease Cancer et al. , 2017). While the effect of socioeconomic factors (SES) on HNC survival has been documented in past literature (Boing et al. 2011; Choi et al. 2016; Johnson et al. 2008), recent studies have started to investigate the effect of area-based socioeconomic deprivation on cancer survival in general (Chang et al. 2012; Rachet et al. 2010; Singh and Jemal 2017), and HNC in particular (Bryere et al. 2017; Chang et al. 2013; Hagedoorn et al. 2016; Megwalu 2017). In Germany, however, studies investigating socioeconomic disparity are scarce and are often limited to certain regions (Brenner et al. 1991; Eberle et al. 2010; Finke et al. 2020; Jansen et al. 2020; Kuznetsov et al. 2011). Jansen et al. published the only large-scale study from Germany that aimed to measure social inequalities in cancer survival in 2014 (Jansen et al. 2014). This study found the 5‐year age‐standardized relative survival of the most deprived patients diagnosed with cancer of the mouth/pharynx to be 45.2% versus 49.3% for the most affluent patients. It is therefore essential to understand the mechanism by which social disparity affects cancer survival and to identify modifiable risk factors.

In this study, we aimed to (1) measure the survival gap according to socioeconomic deprivation level and (2) to decompose the total effect of deprivation on HNC survival into direct effect and indirect effect mediated through other possible factors. To this end, we used population-based and routinely collected data for patients diagnosed with HNC within Germany.

## Materials and methods

### Data source

This retrospective study is based on epidemiological cancer registry data (pooled data from federal registries) from the German Centre for Cancer Registry Data (‘Zentrum für Krebsregisterdaten’, ZfKD) at the Robert Koch Institute (RKI) (Hiripi et al. 2012). The ZfKD annually collects anonymized incidence and survival data from all federal states’ population-based cancer registries. The data then undergo quality checks and are pooled for nationwide and regional analyses. In this analysis, data from the Niedersachsen cancer registry were excluded, as only aggregate socioeconomic data for the entire state (7.9 million inhabitants) were available. Data quality was assessed by proportion of death certificate only (DCO) and autopsy only cases among all registered malignant cancers. Cancer registries were included if the overall proportion of DCO cases in the period 2009–2013 was below 13% as recommended by the European Cancer Registry‐Based Study on Survival and Care of Cancer Patients (EUROCARE‐5 study) (Rossi et al. 2015). Therefore, the final dataset included data from 14 cancer registries covering a population of 69 million people (83% of the total German population).

The German Index of Socioeconomic Deprivation (GISD) was used as a measure for socioeconomic deprivation at the district level (Kroll et al. 2017). Developed by the RKI, the GISD is a composite index that is based on three equally weighted socioeconomic dimensions: income, education, and employment. The income dimension is based on the mean net household income, tax revenues, and debtor quotas within a given district. The educational component is defined by the district’s proportions of employees with (and without) a university degree, school dropouts without a degree, and school dropouts with the German “Abitur” or equivalent. Finally, the employment dimension is measured through the district’s unemployment rate, average gross wage of employees, and the labor force participation rate.

**Fig. 1:**
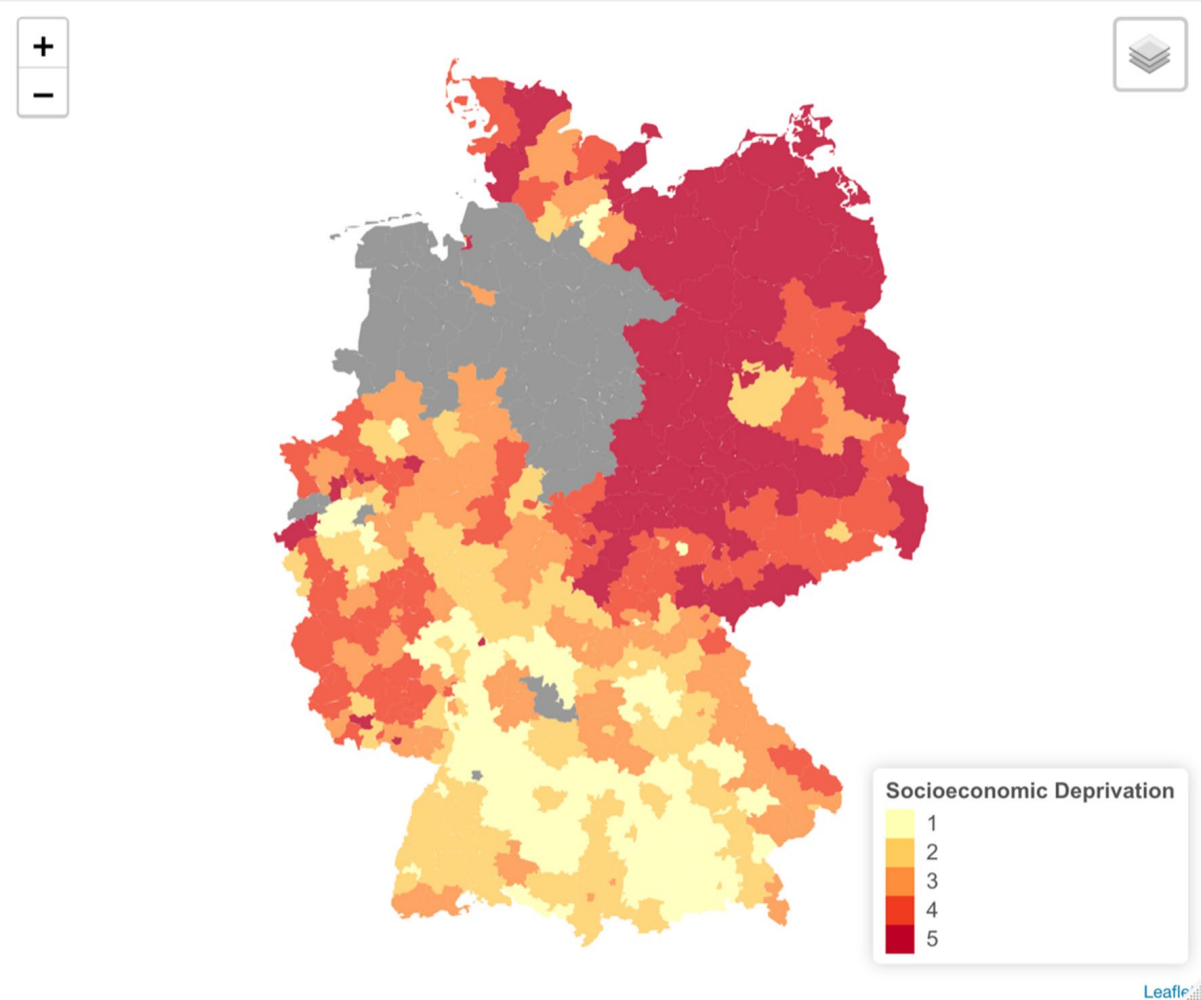
Map of Germany with districts included in the analysis, colored according to their mean level of socioeconomic deprivation, 2009–2013. Quintiles are listed in ascending order according to deprivation (quintile five = most deprived)

The second version of the index, available on GitHub, was used in this analysis (GISD-The German Index of Socioeconomic Deprivation 2020). In the end, 345 districts, out of Germany’s 401 districts, were included in our study after being linked with the pooled registry dataset. We obtained the geo-data for the administrative German districts through the “Bundesamt für Kartographie und Geodäsie (BKG)” website (Bundesamt für Kartographie und Geodäsie (BKG) 2020). Figure 1 shows a map of Germany highlighting the included districts.

### Study population

Our analysis included cases (aged 24–97) diagnosed with malignant squamous cell carcinoma (SCC) in the head and neck region during 2009–2013 and followed up until 31 December 2014. The population-based cancer registries in Germany classify cancer diagnoses based on both the tenth edition of the International Classification of Diseases (ICD-10) and the third edition of the International Classification of Diseases for Oncology (ICD-0–3) (Fritz et al. 2000). Malignant SCC was determined through the morphology codes for squamous cell histology or morphologic variants of SCC (morphology codes: 8032, 8033, 8050–8052, 8070–8078, 8082–8084, 8094, 8123). The included anatomical sites, and their corresponding (ICD-0–3), were: tonsils (C09), base of the tongue (C01.9, C02.4), other oropharynx sites (C10), Waldeyer's ring (C14.2), areas of the oral cavity, gingiva (C03), floor of the mouth (C04), palate (C05), pyriform sinus (C12), and the hypopharynx (C13). Cases of head and neck cancer that could not be distinguished by specific sites were included and grouped as “not specified” (C06).

Carcinoma of unknown primary or recurrent metastasis in the head and neck region of other origin was excluded. In addition, we excluded cases notified by autopsy only or by death certificate only (DCO).

### Exposure and outcome

The exposure under study was the patient’s socioeconomic deprivation level. Each patient’s socioeconomic deprivation level was determined according to the GISD allocated to the case’s district of residence at the time of diagnosis. The indices were then categorized into five quintiles. Quintile one (Q1) represented the least socioeconomically deprived cases while quintile five (Q5) represented the most deprived.

The primary outcome was survival status after cancer diagnosis. For the descriptive analysis and overall survival calculation, survival was treated as a time to event outcome. For the mediation analysis, however, survival was dichotomized (dead vs. alive) and stratified according to time since diagnosis: at 6 months, 1 year conditional on 6-month survival, 2 years conditional on 1-year survival, and 5 years conditional on 2-year survival.

### Covariates

**Fig. 2:**
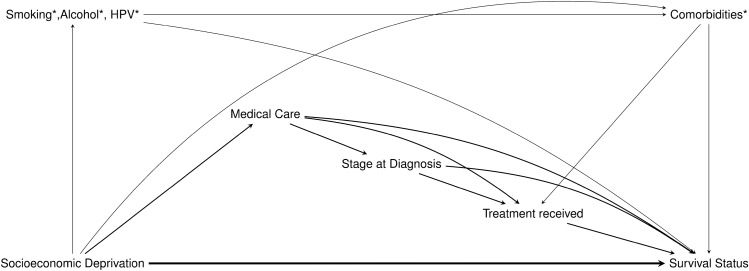
Directed Acyclic Graph (DAG) depicting the causal relationships between deprivation and survival status in HNC patients. Age, sex, and year of diagnosis were considered as baseline confounders

To determine the covariates needed for our analysis and have a better visualization of the causal relationship between them, a directed acyclic graph (DAG) was prepared (Fig. 2). Based on previous research and literature evidence, we assumed that the level of socioeconomic deprivation the patient could experience in his/her district at (the time of diagnosis) could influence the received medical care. In turn, socioeconomic deprivation could influence the patient’s tumor stage at diagnosis and the applied treatment. Thus, while age, gender, and year of diagnosis, were considered as baseline confounders, medical care, stage at diagnosis, and treatment were considered three causally ordered mediators. Our DAG also shows another route that could mediate the effect of deprivation, through smoking, alcohol, and human papillomavirus (HPV) infection. These variables along with comorbidities were not available in our dataset, and therefore considered unmeasured variables.

Medical care was measured as the average number of hospital beds available (at the time of diagnosis) per person, within each district. The number of hospital beds was restricted to those in the oral and maxillofacial, ear-nose-throat (ENT), and radiotherapy departments. This information was available through the Federal Statistical Office (Destatis) registries that are updated annually (Statistisches Bundesamt: Deutsches Krankenhausverzeichnis 2015). Stage at diagnosis was categorized into four groups based on the tumor–node–metastasis (TNM) cancer staging system (Edge and Compton 2010). Information on treatment received was available as four binary variables (surgery yes/no, radiotherapy yes/no, chemotherapy yes/no, immunotherapy yes/no). Based on these variables, we dichotomized treatment into “advanced” and “minor” treatment modes based on what is recommended for each stage by the international guidelines (Network 2020). Only complete cases were included in our analysis.

### Statistical analysis

The observable 5-year overall survival rates (OS) for each quintile were calculated by the Kaplan–Meier method. Multivariate analysis was performed using the Cox proportional hazards model to estimate the hazard ratio (HR) with a 95% confidence interval (CI) for OS. The 5-year age-adjusted relative survival was calculated for each deprivation quintile, as the ratio of observed and expected survival with adjustment to the International Cancer Survival Standards (Corazziari et al. 2004). Expected survival was estimated according to the Ederer II estimator (implemented in the R package “relsurv”) using population life tables stratified by age, sex, and calendar period (Perme and Pavlic 2018).

Mediation analysis, based on the counterfactual framework (Pearl 2013), was then conducted to separate the indirect effects that operate through each of the aforementioned mediators from the remaining direct effect and to quantify their respective contribution towards the overall total effect. We conducted our analysis according to the method proposed by Steen et al. (2017) due to the existence of mediator-outcome confounders that are affected by the exposure and the likely presence of many interactions (VanderWeele et al. 2014). Although this method allows flexible modeling, it still relies on the assumptions of no unaccounted confounding of the exposure–mediator, mediator–outcome or exposure–outcome relationship.

Mediator models were linear (medical care), ordered (stage at diagnosis), or logistic (treatment received) depending on the mediator. The outcome (survival status) was modeled using a logistic model. To obtain a four-way decomposition, we extended our dataset by replicating the observed dataset eight times. We then weighed our extended dataset, by the ratio of densities of the mediators whose corresponding models we believed were less prone to misspecification (medical care and treatment received). An extended version of the outcome model (natural effect model) was then fitted to the original data by regressing imputed nested counterfactuals using our pre-calculated weights. To obtain population-average analogs (rather than effects adjusted on the set of confounders), we updated the weights by inverse weighting. Inverse weighting enables transporting results to the entire target population. Finally, a total of 1,000 bootstrap samples were drawn to calculate 95% (standard normal) bootstrap confidence intervals. This procedure was repeated for each of the previously mentioned time points and only two quintiles were compared at a time. All analyses were conducted in R statistical software version 3.2.3 (Team 2013).

### Sensitivity analysis

To assess the robustness of our findings, we performed different sensitivity analyses. We first explored potential confounding by HPV status. Since this information was not available, we classified HPV status according to tumor site (HPV-related sites vs HPV non-related). This classification was based on studies that found that HPV-positive HNC to be associated with 80% of oropharyngeal HNC and less than 20% of tumors at other anatomic sites of the head and neck (Mehanna et al. 2013). We repeated our Cox regression and mediation analysis while adjusting for this variable.

We also explored potential bias arising from missing treatment and stage information. To have a better understanding regarding variables associated with missing treatment information, we conducted a (forward/backward) stepwise logistic regression.

On the other hand, we assumed missing-stage information to be missing at random (MAR). As a result, we used multiple imputation using chained equations (implemented in the R package “mice”) to impute missing stage (Buuren and Groothuis-Oudshoorn 2010). Our imputation model included all variables from our complete cases dataset. Based on five imputed datasets, we repeated our mediation analysis to include previously excluded patients.

## Results

### Descriptive analysis by deprivation quintiles.

Our analysis included 20,821 cases diagnosed with HNC between 2009 and 2013 from 345 districts in Germany (Table 1). Of the most deprived patients, 48.6% survived up to the end of follow-up, compared to 57.9% of the least deprived patients. Deprived patients were younger and diagnosed at a later stage. Compared to the most affluent (91%), only 79% of the most deprived patients received the advanced treatment according to our definition.

**Table 1:** Characteristics of patients diagnosed with head and neck cancer, 2009–2013

	Deprivation Level						
	All patients		Least Deprived	2	3	4	Most Deprived
Number of patients	20,821		3198	3148	3287	4916	6272
Alive at end of follow-up–no. (%)	10,959 (52.6)		1853 (57.9)	1731 (55.0)	1800 (54.8)	2528 (51.4)	3047 (48.6)
Mean age at diagnosis (SD)	60.9 (10.3)		61.7 (10.4)	61.4 (10.1)	61.7 (10.4)	60.8 (10.2)	59.8 (10.3)
Gender (%)							
Male	77.0		75.5	75.5	74.4	77.4	79.7
Female	23.0		24.5	24.5	25.6	22.6	20.3
Average number of beds^a,b^ (SD)	20.3 (23.1)		23.6 (35.9)	19.1 (17.5)	15.9 (16.5)	21.0 (20.9)	21.1 (21.4)
Stage at Diagnosis (%)							
Stage I	14.5		15.4	14.7	13.3	14.5	14.3
Stage II	11.1		10.2	11.8	11.2	11.1	11.2
Stage III	15.2		14.4	16.4	15.8	14.8	15.0
Stage IV	54.6		53.6	51.3	54.4	55.5	57.6
Missing	4.6		6.2	5.9	5.2	4.0	1.9
Treatment (%)							
Minor	17.1		8.7	13.1	16.7	20.6	20.8
Advanced	82.9		91.3	86.9	83.3	79.4	79.2
Site (%)							
HPV-unrelated	58.2		55.8	58.5	57.0	58.2	60.0
HPV-related	41.8		44.2	41.5	43.0	41.8	40.0

*SD* Standard deviation, *HPV*  Human papillomavirus

^a^The number of hospital beds was restricted to those in the oral and maxillofacial, Ear-Nose-Throat (ENT), and radiotherapy departments

^b^Per 100,000 population

### Overall and standardized survival (net survival)

**Fig. 3:**
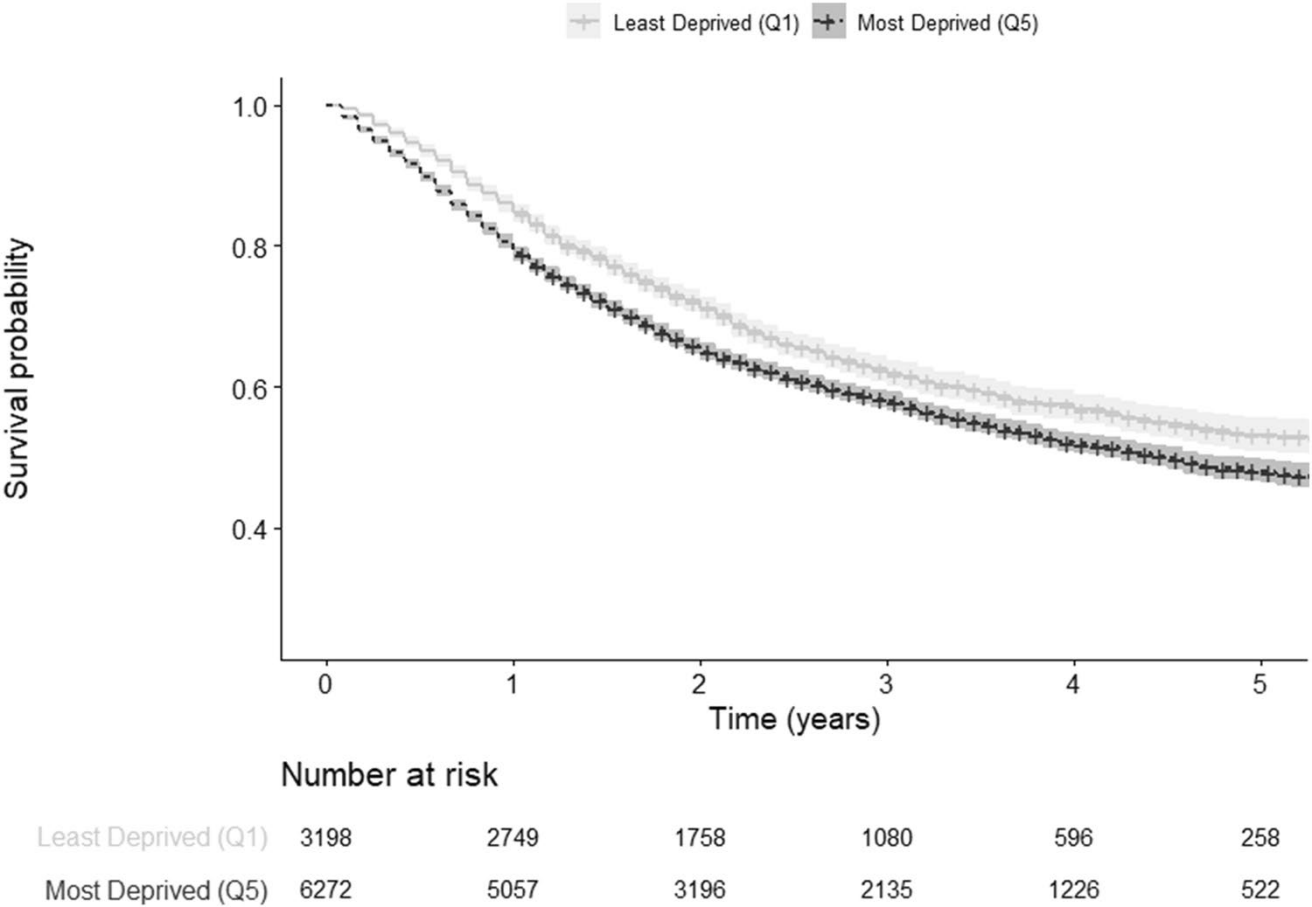
Kaplan–Meier curves comparing survival between least and most socioeconomically deprived patients diagnosed with head and neck cancer in Germany, 2009–2013

The observed 5-year overall survival (OS) for the most affluent patients was 53.2% (95% CI 50.9–55.6). The OS decreased as the level of deprivation increased (51.2, 95% CI 49.0–53.6), (49.1, 95% CI 46.6–51.8), (51.0, 95% CI 49.3–52.8), (47.9, 95% CI 46.3–49.6), for patients in the second, third, fourth, and fifth quintile, respectively (Table 2, Fig. 3).

**Table 2:** Kaplan–Meier, 5-year age-standardized relative survival, and Cox proportional hazards model survival estimates according to deprivation levels of patients diagnosed with head and neck cancer in Germany, 2009–2013

Deprivation quintiles	Kaplan–Meier estimated 5-year overall survival (unadjusted) (95% CI)	5-year age-standardized relative survival (95% CI)	Cox proportional hazards model* Hazard Ratio (95% CI)
Quintile 1	53.2 (50.9–55.6)	56.7 (53.2–59.9)	Reference
Quintile 2	51.2 (49.0–53.6)	56.0 (55.3–60.3)	1.09 (1.01–1.18)
Quintile 3	49.1 (46.6–51.8)	54.0 (50.6–57.3)	1.11 (1.03–1.21)
Quintile 4	51.0 (49.3–52.8)	55.3 (52.8–57.7)	1.13 (1.05–1.21)
Quintile 5	47.9 (46.3–49.6)	50.8 (48.5–53.0)	1.25 (1.17–1.34)

*CI*  confidence interval

^*^Adjusted for age, sex, and year of diagnosis

The 5-year age-standardized survival (net survival) relative to the mortality rates of the general German population showed the first quintile to have the highest relative survival (56.7, 95% CI 53.2–59.9), followed by the second quintile (56.0, 95% CI 55.3–60.3). The fifth quintile still appeared to have the lowest relative survival (50.8, 95% CI 48.5–53.0) (Table 2).

Our Cox regression model, adjusted for age, sex, and year of diagnosis, showed the fifth quintile to have the highest hazard of overall mortality when compared to our reference group (quintile one) ([HR]1.25, 95% CI 1.17–1.34) (Table 2). In the same line, the hazard of overall mortality also increased as the level of deprivation rose. Adjusting for tumor site did not alter the results (Appendix 1).

### Total effect

**Fig. 4:**
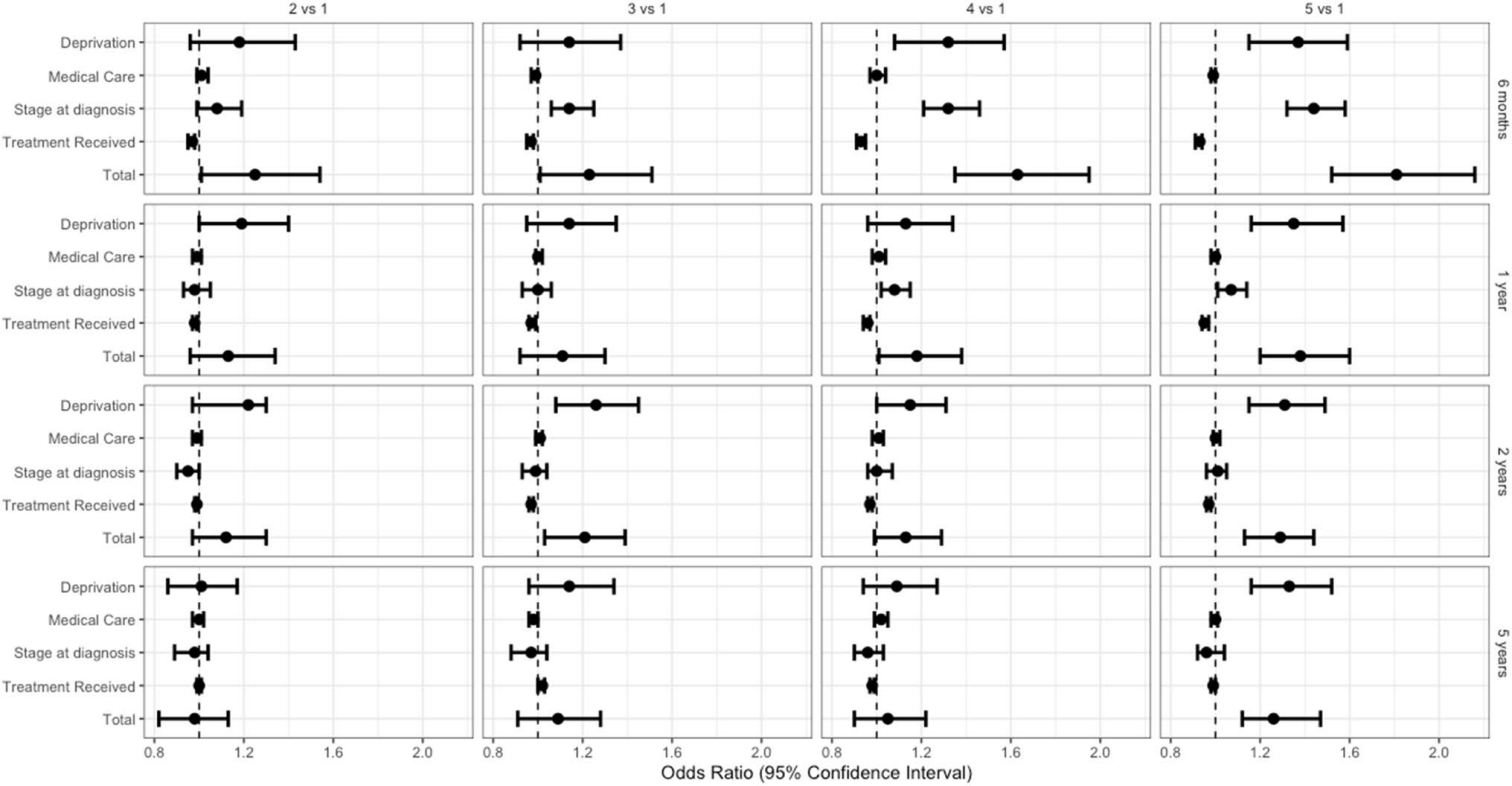
Effect of deprivation and mediators on odds of deaths at different times since head and neck diagnosis

The total effect was defined as the joint effect of deprivation including the indirect effect of the three mediators. The odds of dying were highest during the first 6 months after diagnosis, across all quintiles when compared to the most affluent cases (Odds Ratio [OR] comparing quintile five to quintile one: 1.81, 95% CI 1.52–2.16). Five years after diagnosis (conditioning on 2-year survival), showed that the total effect remained fairly strong only when comparing the most deprived (quintile five) with the least deprived (quintile one) ([OR]: 1.26, 95% CI 1.12–1.47) (Table 3, Fig. 4).

**Table 3:** Effect of Socioeconomic deprivation and mediators on odds of deaths at different times since head and neck diagnosis

	Deprivation level Odds ratio ^a^ (95%CI) (vs reference Q1)				
		Q2	Q3	Q4	Q5
	Direct Effect (SE Deprivation)^b^	1.18 (0.96–1.43)	1.14 (0.92–1.37)	1.32 (1.08–1.57)	1.37 (1.15–1.59)
	Mediator 1 (Medical Care)^c^	1.01 (0.99–1.04)	0.99 (0.97–1.00)	1.00 (0.97–1.04)	0.99 (0.98–1.00)
6 months	M2 (Stage at Diagnosis)^d^	1.08 (0.99–1.19)	1.14 (1.06–1.25)	1.32 (1.21–1.46)	1.44 (1.32–1.58)
	M3 (Treatment)^e^	0.97 (0.95–0.98)	0.97 (0.95–0.98)	0.93 (0.91–0.95)	0.93 (0.91–0.94)
	Total Effect (TE)	**1.25 (1.01–1.54)**	**1.23 (1.01–1.51)**	**1.63 (1.35–1.95)**	**1.81 (1.52–2.16)**
	DE (SE Deprivation)	1.19 (1.00–1.40)	1.14 (0.95–1.35)	1.13 (0.96–1.34)	1.35 (1.16–1.57)
	M1 (Medical Care)	0.99 (0.97–1.01)	1.00 (0.99–1.02)	1.01 (0.98–1.04)	1.00 (0.98–1.01)
1 year*	M2 (Stage at Diagnosis)	0.98 (0.93–1.05)	1.00 (0.93–1.06)	1.08 (1.02–1.15)	1.07 (1.01–1.14)
	M3 (Treatment)	0.98 (0.97–0.99)	0.97 (0.96–0.99)	0.96 (0.94–0.97)	0.95 (0.94–0.97)
	TE	**1.13 (0.96–1.34)**	**1.11 (0.92–1.30)**	**1.18 (1.01–1.38)**	**1.38 (1.20–1.60)**
	DE (SE Deprivation)	1.22 (0.97–1.30)	1.26 (1.08–1.45)	1.15 (1.00–1.31)	1.31 (1.15–1.49)
	M1 (Medical Care)	0.99 (0.97–1.01)	1.01 (0.99–1.02)	1.01 (0.98–1.03)	1.00 (0.99–1.02)
2 years*	M2 (Stage at Diagnosis)	0.95 (0.90–1.00)	0.99 (0.93–1.04)	1.00 (0.96–1.07)	1.01 (0.96–1.05)
	M3 (Treatment)	0.99 (0.98–0.99)	0.97 (0.96–0.98)	0.97 (0.96–0.98)	0.97 (0.96–0.98)
	TE	**1.12 (0.97–1.30)**	**1.21 (1.03–1.39)**	**1.13 (0.99–1.29)**	**1.29 (1.13–1.44)**
	DE (SE Deprivation)	1.01 (0.86–1.17)	1.14 (0.96–1.34)	1.09 (0.94–1.27)	1.33 (1.16–1.52)
	M1 (Medical Care)	1.00 (0.97–1.02)	0.98 (0.96–1.00)	1.02 (0.99–1.05)	1.00 (0.98–1.01)
5 years*	M2 (Stage at Diagnosis)	0.98 (0.89–1.04)	0.97 (0.88–1.04)	0.96 (0.90–1.03)	0.96 (0.92–1.04)
	M3 (Treatment)	1.00 (0.99–1.01)	1.02 (1.00–1.03)	0.98 (0.97–0.99)	0.99 (0.98–1.00)
	TE	**0.98 (0.82–1.13)**	**1.09 (0.91–1.28)**	**1.05 (0.90–1.22)**	**1.26 (1.12–1.47)**

Bold refers to the total effect

*CI* Confidence interval, *Q* Quintile. *SE* Socioeconomic deprivation

^*^Conditional on surviving previous time point

^a^Adjusted for age, sex, and year of diagnosis

^b^The natural direct effect odds ratio of exposure to socioeconomic deprivation levels in different quintiles on odds of death through neither medical care, stage at diagnosis, or treatment

^c^The natural indirect effect odds ratio mediated by exposure induced changes in medical care

^d^The partial indirect effect odds ratio mediated by exposure induced changes in stage at diagnosis

^e^The partial indirect effect odds ratio mediated by exposure induced changes in treatment received

## Indirect effect: role of deprivation and mediators

During the first 6 months after diagnosis, stage at diagnosis seemed to mediate most of the effect of deprivation across the more deprived quintiles. Using a counterfactual reasoning, the odds of dying of the patients in the most affluent quintile would increase by 44% ([OR] 1.44, 95% CI 1.32–1.58) if they were to be diagnosed as patients in quintile five (while keeping their level of deprivation, medical care, and treatment received unchanged and adjusting for age, sex, and year of diagnosis).

One year after diagnosis, the mediated effect of differential stage at diagnosis is only apparent in the fourth and fifth quintile. As follow-up time increases, there was no evidence that the considered mediators could contribute to the effect of deprivation on survival. Medical care and differential treatment seem to play no relevant role in mediating the effect of deprivation on survival (Table 3, Fig. 4).

Including tumor site as a confounder or including imputed stage information did not alter our results (Appendix 1, 2).

## Discussion

Patients living in the most deprived districts at the time of diagnosis, showed the lowest survival rates according to our analysis. The total effect of deprivation seemed to be strongest during the first six months after diagnosis. While the effect subsided considerably at later time points, the survival disparity between the most deprived and most affluent remained substantial after 5 years. Our mediation analysis showed that stage at diagnosis played a major role in mediating the effect of deprivation within the first 6 months after diagnosis. Its role diminishes, however, as follow-up time increases. In contrast, there was no evidence that treatment and medical care mediated any of the effect of deprivation on survival throughout the study period.

Given that our study is based on a large sample size drawn from the national cancer registry, our results confirmed the survival disparity between the deprived and affluent patients in Germany, which is in line with Jansen et al. (Jansen et al. 2014). This survival gap, however, is difficult to explain in light of the universal health care system present.

To our knowledge, this is the first study that employs a counterfactual causal inference approach to gain a comprehensive understanding of the direct and mediated effect of social disparity on HNC survival in Germany. Through our DAG, we presented a detailed framework to analyze causal relations and to identify potential factors that could help explain the effect of socioeconomic deprivation. By having a clear visualization of the causal relations among variables, we were able to avoid potential biases (such as indication bias or selection bias), which could arise, for example, from the medical care-comorbidities-treatment relationship.

Based on the current literature available, we presented three potential mediators: medical care, stage at diagnosis, and treatment. Medical care for instance, was included as a mediator in our analysis based on the inequalities in health care utilization and availability experienced in Germany (Geyer 2008; Klein and von dem Knesebeck 2016). Patients from lower socioeconomic groups have been found to visit specialist practitioners less frequently, when compared with groups that are more affluent (Gruber 2010). Furthermore, results from a systematic review by Klein et al., suggested that major inequalities result primarily from prevention strategies, such as cancer screening (Klein et al. 2013).

Remarkably, in a study that investigated the effect of deprivation on breast cancer survival, Li et al. found that 35% (23–48%) of the higher mortality experienced by most deprived patients at six months after breast cancer diagnosis, was mediated by adverse stage distribution (Li et al. 2016). While stage at diagnosis is already recognized as a major prognostic factor in cancer survival, these results are interesting considering the wide availability of an advanced health care system in the UK, which is comparable to Germany.

Medical care along with minor vs. advanced treatment, on the other hand, revealed no evidence in mediating the effect of deprivation. Since the standardized “quality of health care” index is not available on a district level, we included the number of hospital beds (in the three previously mentioned departments) per districts’ population as an indicator of health-care availability and access. Information, like health insurance coverage status (private vs public) or waiting times however, were not available in our measurement. In a study by Lungen et al., patients covered by the statutory health insurance (public option) were found to wait 3.08 times longer for an appointment than private health insurees in Germany (Lungen et al. 2008). Lacking this information could have led to the underestimation of the mediated effects of these factors. Moreover, missing-stage information could have also played a significant role in this regard. A large proportion of missing treatment information (49.3%) was linked to patients living in the most affluent districts (Appendix 2). This was confirmed by our stepwise logistic regression that revealed deprivation level, age, medical care, and stage as the most significantly associated variables to missing treatment information (Appendix 2). In contrast, only a small percentage of stage information was missing (4.6%).

From a clinical perspective, it seems surprising that treatment fails to mediate the mentioned effects. This could be explained by that treatment cannot compensate for the adverse survival prospect due to an advanced stage. However, in our analysis, we could not fully account for details of the treatment, such as the intent of treatment, administered radiation dose, the chemotherapy given, or the surgical procedure performed. Treatment in the form defined seems to be universally available and might follow the average health performance in a district that determines the received treatment.

Considering that the development of HNC is a multifactorial process associated with a variety of risk factors, we have also presented an alternate route in our DAG that could also explain the effect of deprivation on survival. Major risk factors that were missing in our dataset, such as tobacco, alcohol consumption, and comorbidities have already been established as prognostic variables that are directly influenced by socioeconomic factors. In addition, HPV infections have been recently linked to up to 25% of HNC cases (Kreimer et al. 2005). Patients diagnosed with HPV-positive HNC were more likely to be younger men, non-smokers, and have higher SES when compared with HPV-negative HNC patients (Gillison et al. 2008). HPV-positive oropharyngeal carcinoma is also associated with better response to treatment and better survival (Ang et al. 2010; O’Rorke et al. 2012). It was, therefore, necessary to address potential bias that might arise from the missing HPV status. The pathologic evaluation of HPV status is currently based on PCR-based strategies, type-specific in situ hybridization(ISH) techniques, and immune-histochemical detection of surrogate biomarkers (e.g. p16 protein) (Westra 2009). Tumors positive both for p16 immunochemistry and HPV ISH are usually classified as HPV-positive (Robinson et al. 2010). While acknowledging this as a limitation, we performed our sensitivity analysis based on tumor site, which we considered a proxy for the missing HPV status. We found no significant differences in tumor-site proportions according to deprivation, nor did our results change when we included tumor site as an additional confounder in our Cox regression and mediation analysis.

## Conclusion

Our results confirmed the survival gap between deprived and affluent patients in Germany. We were able to quantify the direct effect of socioeconomic deprivation on survival and the effect mediated through medical care, stage at diagnosis, and treatment received. Considering data limitations, our results suggest that elimination of disparities in stage at diagnosis could contribute to a substantial reduction in survival disparities.

## Supplementary Information

Below is the link to the electronic supplementary material.

Appendix 1: Treating tumor site as a confounder: sensitivity analysis (PDF 440 KB)

Appendix 2: Missing data: sensitivity analysis (PDF 401 KB)

Appendix 3: R code for mediation analysis (PDF 278 KB)

## Data Availability

This study was based on the German national cancer registry data. The authors do not own these data and hence are not permitted to share them in the original form (only in aggregate form, e. g, publications). Data were provided by the Robert Koch Institute (RKI).

## Code availability

Code is available in Appendix 3.
