# Supplementary material for: Socioeconomic disparities in head and neck cancer survival in Germany: a causal mediation analysis using population-based cancer registry data
Source: J Cancer Res Clin Oncol. 2021 Feb 11;147(5):1325–34. doi: 10.1007/s00432-021-03537-2 (PMC8021523; doi:10.1007/s00432-021-03537-2)
Supplement: Supplementary file 1 — Appendix 1: Treating tumor site as a confounder: sensitivity analysis (PDF 440 KB) [file 432_2021_3537_MOESM1_ESM.pdf]

Socioeconomic disparities in head and neck cancer survival in Germany: A causal mediation analysis using population-based cancer registry data.

Bedir, Ahmed[1]; Abera, Semaw Ferede[1]; Efremov, Ljupcho[1,2]; Hassan, Lamiaa[2]; Vordermark, Dirk[1,3]; Medenwald, Daniel[1,3]

1. Department of Radiation Oncology, Health Services Research Group, University Hospital Halle (Saale), Ernst-Grube-Str. 40, 06120, Halle (Saale), Germany.
2. Institute of Medical Epidemiology, Biometry, and Informatics, Martin Luther University Halle-Wittenberg, Magdeburger Strasse 8, 06112, Halle (Saale), Germany
3. Department of Radiation Oncology, University Hospital Halle (Saale), Ernst-Grube-Str. 40, 06120, Halle (Saale), Germany.

Address for correspondence:
Daniel Medenwald
Department of Radiation Oncology, University Hospital Halle (Saale),
Ernst-Grube-Str. 40, 06120, Halle (Saale), Germany.
Telephone no: +49-345-557-3453/4027
Email: Daniel.Medenwald@uk-halle.de

Appendix 1: Treating Tumor Site as a confounder: Sensitivity Analysis.

Table 1. Cox proportional hazards model analyses.

**Deprivation Quintile**<br>*(vs Reference Quintile 1)*	**Hazard Ratio***	**95% CI**
Quintile 2	1.08	1.00-1.17
Quintile 3	1.11	1.03-1.20
Quintile 4	1.12	1.04-1.21
Quintile 5	1.24	1.16-1.33

*Adjusted for age, sex, year of diagnosis, and tumor site.

Abbreviations: CI=Confidence interval.

Table 2. Effect of Socioeconomic (SE) deprivation and mediators on odds of deaths at different times since head and neck diagnosis including tumor site as an additional confounder.

		**Deprivation Level Odds Ratio [a] (95%CI) (vs reference Q1)**			
		**Q2**	**Q3**	**Q4**	**Q5**
**6 months**	Direct Effect (SE Deprivation)[a]	1.17 (0.94-1.42)	1.14 (0.92-1.38)	1.32 (1.09-1.57)	1.37 (1.14-1.64)
	Mediator 1 (Medical Care)[b]	1.02 (0.99-1.04)	0.99 (0.97-1.00)	1.00 (0.97-1.04)	0.99 (0.98-1.00)
	M2 (Stage at Diagnosis)[c]	1.07 (0.98-1.19)	1.14 (1.04-1.26)	1.32 (1.22-1.45)	1.45 (1.33-1.58)
	M3 (Treatment)[d]	0.97 (0.95-0.98)	0.96 (0.95-0.98)	0.93 (0.91-0.95)	0.92 (0.90-0.94)
	Total Effect (TE)	**1.24 (0.99-1.53)**	**1.23 (1.00-1.51)**	**1.63 (1.35-1.95)**	**1.81 (1.51-2.16)**
**1 year***	DE (SE Deprivation)	1.18 (0.99-1.39)	1.14 (0.96-1.34)	1.13 (0.96-1.32)	1.34 (1.17-1.56)
	M1 (Medical Care)	0.99 (0.97-1.01)	1.00 (0.99-1.02)	1.01 (0.98-1.04)	1.00 (0.98-1.01)
	M2 (Stage at Diagnosis)	0.98 (0.93-1.05)	0.99 (0.93-1.07)	1.08 (1.01-1.15)	1.08 (1.01-1.15)
	M3 (Treatment)	0.98 (0.97-0.99)	0.97 (0.96-0.99)	0.95 (0.94-0.97)	0.95 (0.94-0.96)
	TE	**1.12 (0.95-1.33)**	**1.10 (0.93-1.30)**	**1.17 (1.00-1.37)**	**1.37 (1.19-1.59)**
**2 years***	DE (SE Deprivation)	1.21 (0.99-1.38)	1.25 (1.09-1.43)	1.14 (0.98-1.31)	1.30 (1.14-1.48)
	M1 (Medical Care)	0.99 (0.97-1.01)	1.01 (1.00-1.02)	1.01 (0.98-1.04)	1.00 (0.99-1.02)
	M2 (Stage at Diagnosis)	0.94 (0.88-0.99)	0.98 (0.92-1.05)	1.00 (0.95-1.07)	1.01 (0.95-1.05)
	M3 (Treatment)	0.98 (0.98-0.99)	0.97 (0.95-0.98)	0.97 (0.96-0.98)	0.96 (0.95-0.97)
	TE	**1.11 (0.95-1.27)**	**1.20 (1.04-1.37)**	**1.11 (0.96-1.28)**	**1.27 (1.10-1.43)**
**5 years***	DE (SE Deprivation)	1.00 (0.85-1.17)	1.14 (0.98-1.33)	1.09 (0.94-1.26)	1.33 (1.15-1.51)
	M1 (Medical Care)	1.00 (0.97-1.02)	0.98 (0.96-1.00)	1.02 (0.99-1.05)	1.00 (0.98-1.01)
	M2 (Stage at Diagnosis)	0.97 (0.89-1.03)	0.96 (0.87-1.04)	0.96 (0.90-1.04)	0.96 (0.91-1.05)
	M3 (Treatment)	1.00 (0.99-1.01)	1.01 (1.00-1.03)	0.98 (0.97-0.99)	0.99 (0.98-1.00)
	TE	**0.97 (0.81-1.12)**	**1.09 (0.92-1.27)**	**1.05 (0.90-1.23)**	**1.26 (1.11-1.47)**

a Adjusted for age, sex, and year of diagnosis and tumor site.

b The natural direct effect odds ratio of exposure to socioeconomic deprivation levels in different quintiles on odds of death through neither medical care, stage at diagnosis, or treatment.

c The natural indirect effect odds ratio mediated by exposure induced changes in medical care.

d The partial indirect effect odds ratio mediated by exposure induced changes in stage at diagnosis.

e The partial indirect effect odds ratio mediated by exposure induced changes in treatment received.

* Conditional to surviving previous time point. Abbreviations: CI= Confidence interval, Q= Quintile. SE= Socioeconomic deprivation
