# Supplementary material for: Socioeconomic disparities in head and neck cancer survival in Germany: a causal mediation analysis using population-based cancer registry data
Source: J Cancer Res Clin Oncol. 2021 Feb 11;147(5):1325–34. doi: 10.1007/s00432-021-03537-2 (PMC8021523; doi:10.1007/s00432-021-03537-2)
Supplement: Supplementary file 2 — Appendix 2: Missing data: sensitivity analysis (PDF 401 KB) [file 432_2021_3537_MOESM2_ESM.pdf]

Socioeconomic disparities in head and neck cancer survival in Germany: A causal mediation analysis using population-based cancer registry data.

Bedir, Ahmed[1]; Abera, Semaw Ferede[1]; Efremov, Ljupcho[1,2]; Hassan, Lamiaa[2]; Vordermark, Dirk[1,3]; Medenwald, Daniel[1,3]

1. Department of Radiation Oncology, Health Services Research Group, University Hospital Halle (Saale), Ernst-Grube-Str. 40, 06120, Halle (Saale), Germany.
2. Institute of Medical Epidemiology, Biometry, and Informatics, Martin Luther University Halle-Wittenberg, Magdeburger Strasse 8, 06112, Halle (Saale), Germany
3. Department of Radiation Oncology, University Hospital Halle (Saale), Ernst-Grube-Str. 40, 06120, Halle (Saale), Germany.

Address for correspondence:
Daniel Medenwald
Department of Radiation Oncology, University Hospital Halle (Saale),
Ernst-Grube-Str. 40, 06120, Halle (Saale), Germany.
Telephone no: +49-345-557-3453/4027
Email: Daniel.Medenwald@uk-halle.de

Appendix 2: Missing Data: Sensitivity Analysis

Table 1. Proportion of missing information of stage at diagnosis and treatment received.

		Deprivation Level				
	All patients	Least Deprived	2	3	4	Most Deprived
**Stage at Diagnosis (%)**						
Stage I	14.5	15.4	14.7	13.3	14.5	14.3
Stage II	11.1	10.2	11.8	11.2	11.1	11.2
Stage III	15.2	14.4	16.4	15.8	14.8	15.0
Stage IV	54.6	53.6	51.3	54.4	55.5	57.6
Missing	4.6	6.2	5.9	5.2	4.0	1.9
**Treatment (%)**						
Minor	13.1	5.0	8.5	13.5	18.5	19.5
Advanced	58.1	45.7	49.4	58.4	63.4	71.8
Missing	28.8	49.3	42.1	28.1	18.2	8.6

Table 2. Logistic regression showing association of missing treatment information and age and deprivation quintile and their interactions.

Coefficients	Odds Ratio	95% CI	P-value
Age	1.01	1.00-1.02	<0.001
Quintile 1	Reference	---	----
Quintile 2	0.73	0.46-1.14	0.16
Quintile 3	0.74	0.45-1.21	0.24
Quintile 4	0.20	0.12-0.33	<0.001
Quintile 5	0.07	0.04-0.12	<0.001
Age*Quintile 2	1.00	0.99-1.01	0.88
Age*Quintile 3	0.99	0.98-1.00	0.02
Age*Quintile 4	1.00	0.99-1.01	0.51
Age*Quintile 5	1.00	1.00-1.01	0.13

* Interaction

Abbreviations: CI=Confidence interval.

Table 3. Stepwise logistic regression to determine variables that were associated the most with missing treatment information.

Coefficients	Odds Ratio	95% CI	P-value
Age	1.01	1.00-1.01	<0.001
Sex (male)	0.95	0.89-1.02	0.14
Quintile 1	Reference	---	---
Quintile 2	0.73	0.68-0.79	<0.001
Quintile 3	0.39	0.36-0.43	<0.001
Quintile 4	0.23	0.21-0.25	<0.001
Quintile5	0.10	0.09-0.11	<0.001
Stage I	Reference	---	---
Stage II	0.78	0.71-0.87	<0.001
Stage III	0.58	0.52-0.63	<0.001
Stage IV	0.54	0.50-0.59	<0.001
Medical care	0.99	0.98-1.00	<0.001

Abbreviations: CI=Confidence interval.

Table 4. Effect of Socioeconomic deprivation and mediators on odds of deaths at different times since head and neck diagnosis based on five imputed datasets.

		Deprivation Level Odds Ratio [a] (95%CI) (vs reference Q1)			
		**Q2**	**Q3**	**Q4**	**Q5**
**6 months**	DE (SE Deprivation)[a]	1.18 (1.10-1.28)	1.14 (1.05-1.22)	1.31 (1.23-1.40)	1.36 (1.28-1.44)
	M1 (Medical Care)[b]	1.02 (1.01-1.03)	0.99 (0.98-1.00)	1.00 (0.99-1.02)	0.99 (0.98-0.99)
	M2 (Stage at Diagnosis)[c]	1.09 (1.06-1.13)	1.14 (1.11-1.19)	1.32 (1.27-1.37)	1.42 (1.38-1.47)
	M3 (Treatment)[d]	0.96 (0.95-0.97)	0.96 (0.95-0.97)	0.93 (0.92-0.94)	0.93 (0.92-0.93)
	Total Effect (TE)	**1.28 (1.18-1.39)**	**1.24 (1.14-1.34)**	**1.60 (1.50-1.72)**	**1.76 (1.65-1.88)**
**1 year***	DE (SE Deprivation)	1.21 (1.13-1.29)	1.14 (1.07-1.22)	1.14 (1.07-1.23)	1.36 (1.29-1.44)
	M1 (Medical Care)	0.99 (0.98-1.00)	1.00 (1.00-1.01)	1.01 (1.00-1.02)	1.00 (0.99-1.01)
	M2 (Stage at Diagnosis)	0.98 (0.96-1.00)	1.01 (0.99-1.04)	1.09 (1.06-1.12)	1.06 (1.04-1.09)
	M3 (Treatment)	0.98 (0.97-0.98)	0.97 (0.96-0.97)	0.95 (0.94-0.96)	0.96 (0.95-0.96)
	TE	**1.16 (1.08-1.23)**	**1.12 (1.05-1.20)**	**1.19 (1.13-1.28)**	**1.38 (1.31-1.46)**
**2 years***	DE (SE Deprivation)	1.24 (1.17-1.31)	1.25 (1.18-1.33)	1.15 (1.08-1.21)	1.34 (1.29-1.41)
	M1 (Medical Care)	0.99 (0.98-1.00)	1.00 (1.00-1.01)	1.01 (1.00-1.02)	1.00 (1.00-1.01)
	M2 (Stage at Diagnosis)	0.95 (0.94-0.98)	0.99 (0.96-1.00)	0.99 (0.97-1.02)	1.01 (0.99-1.02)
	M3 (Treatment)	0.98 (0.98-0.99)	0.97 (0.96-0.97)	0.97 (0.96-0.97)	0.97 (0.96-0.97)
	TE	**1.15 (1.08-1.21)**	**1.20 (1.12-1.27)**	**1.12 (1.06-1.18)**	**1.32 (1.25-1.37)**
**5 years***	DE (SE Deprivation)	1.01 (0.95-1.08)	1.10 (1.04-1.18)	1.07 (1.00-1.13)	1.30 (1.24-1.37)
	M1 (Medical Care)	1.00 (0.99-1.01)	0.98 (0.97-0.99)	1.02 (1.01-1.03)	1.00 (0.99-1.00)
	M2 (Stage at Diagnosis)	0.99 (0.96-1.02)	0.99 (0.96-1.02)	0.96 (0.93-0.99)	0.95 (0.93-0.98)
	M3 (Treatment)	1.00 (0.99-1.00)	1.01 (1.01-1.02)	0.98 (0.97-0.98)	0.99 (0.99-1.00)
	TE	**1.00 (0.94-1.07)**	**1.08 (1.02-1.16)**	**1.03 (0.96-1.09)**	**1.23 (1.17-1.29)**

a Adjusted for age, sex, and year of diagnosis.
b The natural direct effect odds ratio of exposure to socioeconomic deprivation levels in different quintiles on odds of death through neither medical care, stage at diagnosis, or treatment.
c The natural indirect effect odds ratio mediated by exposure induced changes in medical care.
d The partial indirect effect odds ratio mediated by exposure induced changes in stage at diagnosis.
e The partial indirect effect odds ratio mediated by exposure induced changes in treatment received.
* Conditional to surviving previous time point.
Abbreviations: CI= Confidence interval, Q= Quintile. SE= Socioeconomic deprivation
