# Supplementary material for: Socioeconomic disparities in head and neck cancer survival in Germany: a causal mediation analysis using population-based cancer registry data
Source: J Cancer Res Clin Oncol. 2021 Feb 11;147(5):1325–34. doi: 10.1007/s00432-021-03537-2 (PMC8021523; doi:10.1007/s00432-021-03537-2)
Supplement: Supplementary file 3 — Appendix 3: R code for mediation analysis (PDF 278 KB) [file 432_2021_3537_MOESM3_ESM.pdf]

Socioeconomic disparities in head and neck cancer survival in Germany: A causal mediation analysis using population-based cancer registry data.

Bedir, Ahmed[1]; Abera, Semaw Ferede[1]; Efremov, Ljupcho[1,2]; Hassan, Lamiaa[2]; Vordermark, Dirk[1,3]; Medenwald, Daniel[1,3]

1. Department of Radiation Oncology, Health Services Research Group, University Hospital Halle (Saale), Ernst-Grube-Str. 40, 06120, Halle (Saale), Germany.
2. Institute of Medical Epidemiology, Biometry, and Informatics, Martin Luther University Halle-Wittenberg, Magdeburger Strasse 8, 06112, Halle (Saale), Germany
3. Department of Radiation Oncology, University Hospital Halle (Saale), Ernst-Grube-Str. 40, 06120, Halle (Saale), Germany.

Address for correspondence:
Daniel Medenwald
Department of Radiation Oncology, University Hospital Halle (Saale),
Ernst-Grube-Str. 40, 06120, Halle (Saale), Germany.
Telephone no: +49-345-557-3453/4027
Email: Daniel.Medenwald@uk-halle.de

Appendix 3: R Code for Mediation Analysis.

1. We first fitted a linear regression for the first mediator (m1) (medical care “loghosbeds”) and a logistic regression for the third mediator (m3) (treatment “tx”) conditional on the exposure (socioeconomic deprivation level “SD”), potential earlier intermediates (Stage at diagnosis “stage_dx”), and our confounders (age, sex, and year of diagnosis).

```
  fitm1<-glm(loghosbeds~factor(SD)+ age+ sex+ diagyear, family = gaussian("identity"), data = hanm1)

  fitm3<-glm(tx~factor(SD)*loghosbeds*factor(stage_dx)+ age+ sex+ diagyear,family = binomial, data = hanm1)
```

2. We then fitted a logistic regression for the outcome variable (death at 6 months, 1 year conditional on 6-month survival, 2 years conditional on 1-year survival, or 5 years conditional on 2-year survival “TOD”).

```
fitY<-glm(TOD~factor(SD)*loghosbeds*factor(stage_dx)*factor(tx)+ age+ sex+ diagyear, family = binomial, data = hanm1)
```

3. We then extended our original dataset 8 times. Considering that the exposure levels analyzed at any one time were 2 (most affluent vs any other level) and we had 3 mediators (2^3=8). The extended dataset also included hypothetical exposure levels: a1, a2, and a3.

```
expData<-data.frame(replicate= rep(1:8, times =nrow(hanm1)), hanm1[rep(hanm1$id, each = 8), ], a0=NA, a1=NA, a2=NA,a3=NA)

expData<-within(expData, {

  a2 <- A

  a0 <- ifelse(replicate %in% c(2,4,6,8), 1-A, A)

  a1 <- ifelse(replicate %in% c(3,4,7,8), 1-A, A)

  a3 <- ifelse(replicate %in% c(5,6,7,8), 1-A, A)

})
```

4. We then calculated regression weights.

```
num1 <- with(expData, dnorm(loghosbeds, mean = predict(fitm1, newdata = within(expData, A <- a1), type = "response"), sd = sqrt(summary(fitm1)$dispersion)))

num2 <- with(expData, predict(fitm3, newdata = within(expData, A <- a3),type = "response"))

denom1 <- with(expData, dnorm(loghosbeds, mean = predict(fitm1, newdata = within(expData, A <- a2), type = "response"), sd = sqrt(summary(fitm1)$dispersion)))

denom2 <- with(expData,predict(fitm3, newdata = within(expData, A <- a2), type = "response"))

expData$W1 <- (num1*num2)/(denom1*denom2)
```

5. To calculation population-average component effects, we update the previously calculated weights by inverse weighting.

```
fitA <- glm(SD~age+ sex+ diagyear, family = binomial("logit"), data=hanm1)

expData<-within (expData, {W1<-W1 / dbinom(A, size = 1, prob = predict(fitA,
newdata=expData, type="response"))})
```

6. We finally fit a population-average natural effect model.

```
fitNEMpop<- glm(TOD ~ a0*a1*a2*a3 , family=binomial("logit"), data = expData, weights =
W1)
```

The analysis was repeated for each of the previously mentioned time points and only two quintiles were compared at a time.
